# Supplementary material for: Compounds producing an effective combinatorial regimen for disruption of HIV‐1 latency
Source: EMBO Mol Med. 2017 Dec 15;10(2):160–74. doi: 10.15252/emmm.201708193 (PMC5838563; doi:10.15252/emmm.201708193)
Supplement: Supplementary file 1 — Appendix [file EMMM-10-160-s001.pdf]

Appendix Figures and Tables

Compounds Producing an Effective Combinatorial Regimen for Disruption of HIV-1 Latency

Pargol Hashemi[1], Kris Barreto[1], Wendy Bernhard[1], Adam Lomness[1], Nicolette Honson[2], Tom A. Pfeifer[2], P. Richard Harrigan[3], and Ivan Sadowski[1]*

[1]Biochemistry and Molecular Biology, Molecular Epigenetics, Life Sciences Institute, University of British Columbia, Vancouver, BC V6T1Z3; Canada.
[2]The Centre for Drug Research and Development; Vancouver, BC V6T 1Z3; Canada.
[3]BC Centre for Excellence in HIV/AIDS, St. Paul's Hospital, Vancouver, BC V6Z 1Y6; Canada.

Table of Content

Appendix Figure	Page
Appendix Figure S1	2
Appendix Figure S2	3
Appendix Figure S3	4
Appendix Figure S4	5
Appendix Figure S5	6
Appendix Table S1	7-11
Appendix Table S2	12
Appendix Table S3	13
Appendix Table S4	14
Appendix Table S5	15

Appendix Figure S1

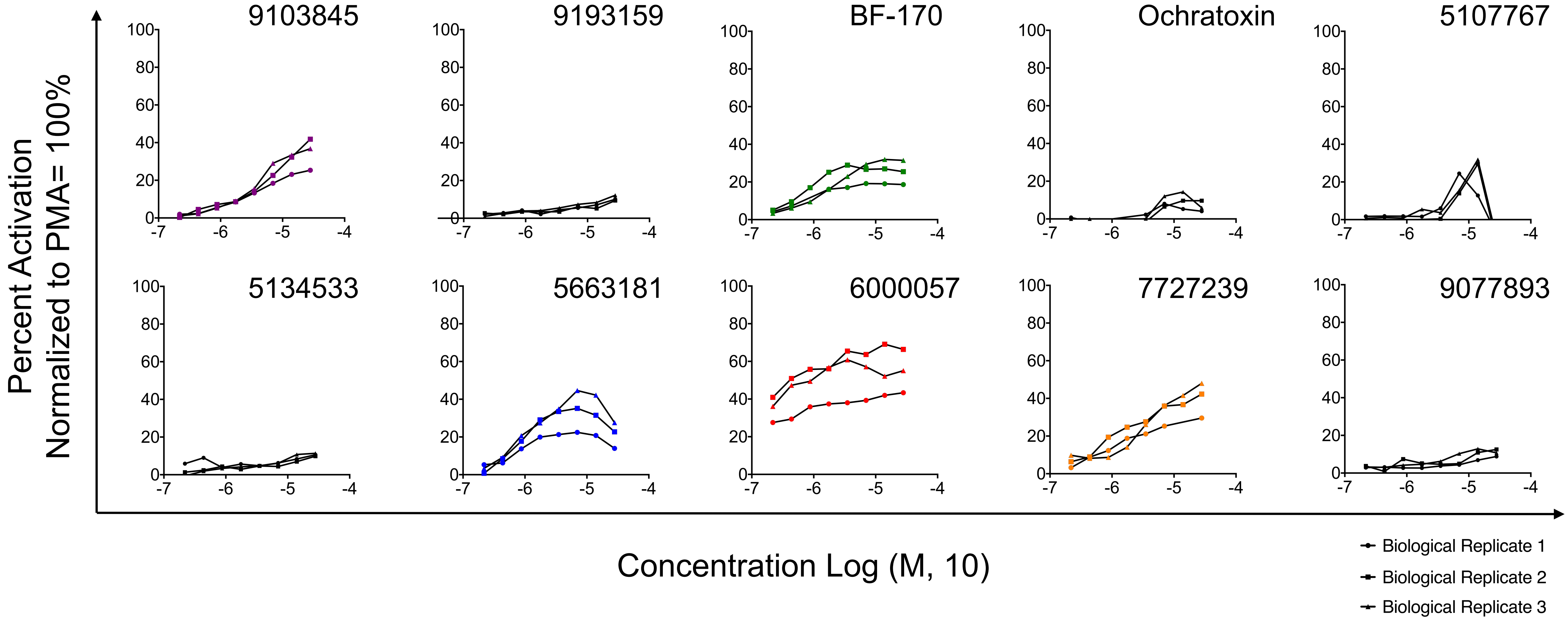

Appendix Figure S1. Re-examining ten compounds in the secondary assays. The compounds from the initial HTS of the three libraries that produced the strongest GFP expression were re-assayed in the Jurkat^{Tat} LTR-luciferase cell line. The luciferase expression was measured after 24 h of treatment and presented as a percent activation relative to the results from the cells treated with PMA. Among the ten compounds represented, along with their corresponding company ID number, five (illustrated with colored dots) showed significant activity toward reversing latency in a dose-dependent manner. As table 1 summarizes, these five compounds are designated as PH01-PH05. Results are determined from three biological replicates (n=3).

Appendix Figure S2

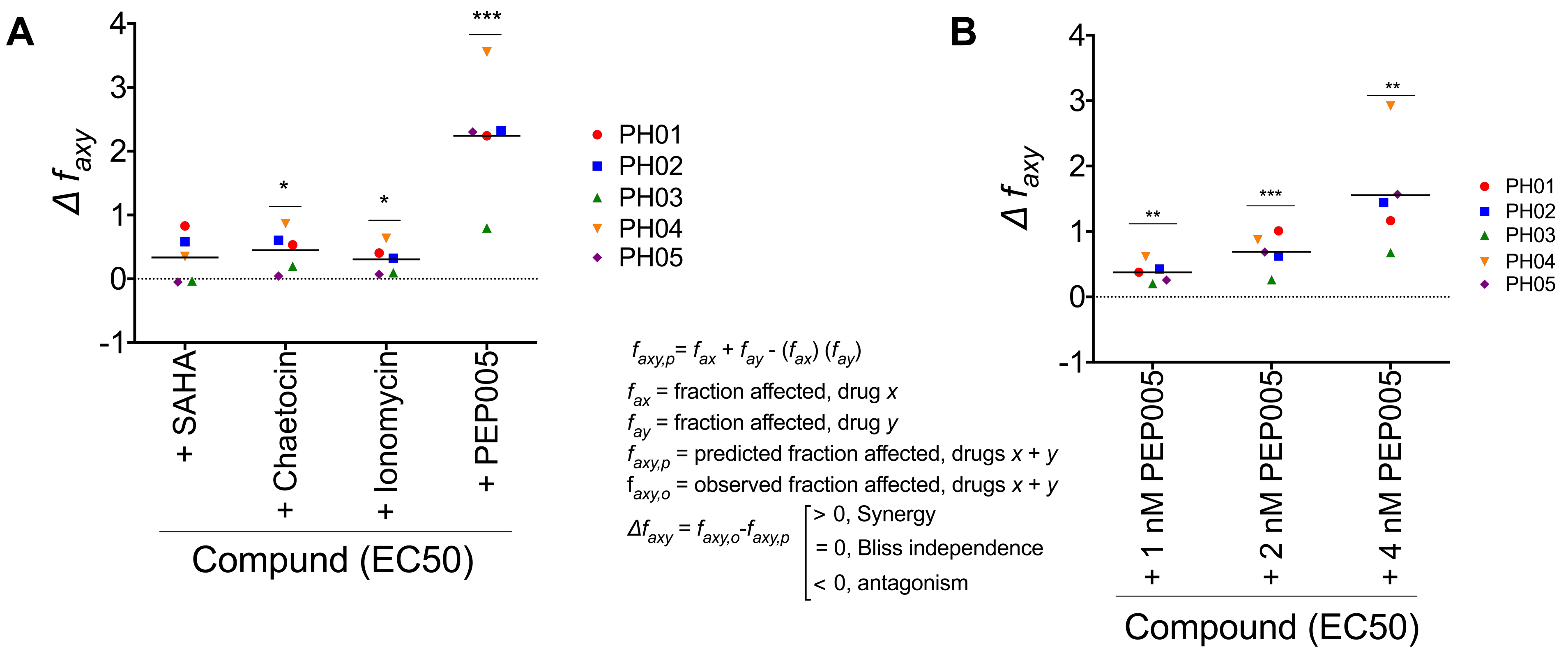

Appendix Figure S2. Statistical analysis assessing the latency-reversing activity of drug combinations.

A Bliss independence determinations were calculated for results from treatment with PH01 - PH05 in combination with SAHA, Chaetocin, Ionomycin, and PEP005.

B The Bliss independence model was applied to calculate Δf_{axy} associated with PH compounds (EC50) in combination with the indicated PEP005 concentrations.

Data information: Statistical significance, derived from ratio paired t test analysis is indicated as: *, $P<0.05$; **, $P<0.005$; ***, $P<0.0005$; ****. The exact P-values are indicated in the Appendix Table S1.

Appendix Figure S3

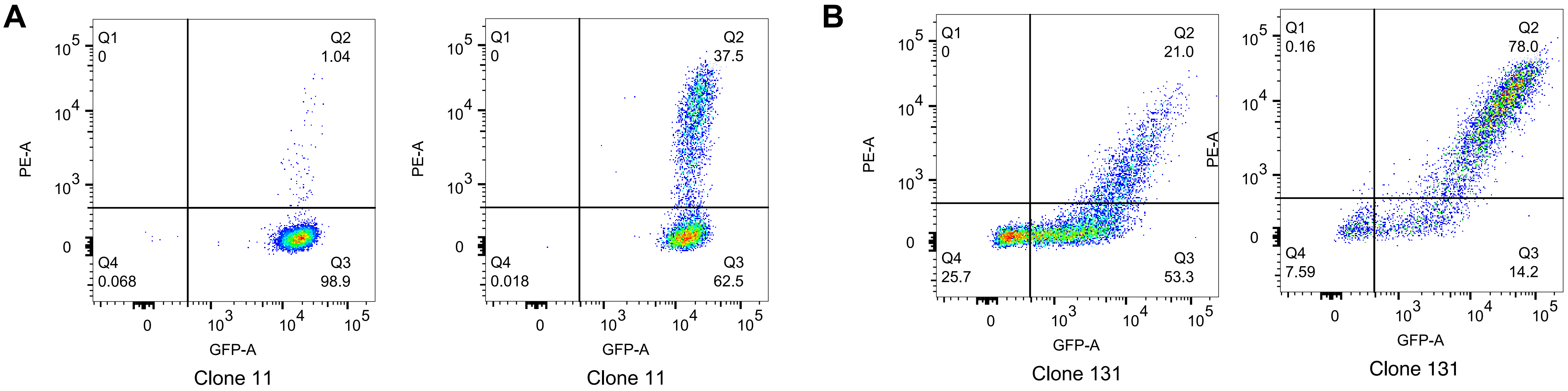

Appendix Figure S3. Fluorescence-activated cell sorting (FACS) of cells bearing md-HIV provirus indicates expression of EIF-1α-GFP and LTR-dsRed. Stimulation of the md-HIV lines generally causes a shift of the fluorescence profile from GFP+/DsRed- toward GFP+/DsRed+.

A Jurkat^{Tat} LTR-DsRed, Clone # 11: unstimulated cells (left panel) and PMA-stimulated cells (right panel).

B Jurkat^{Tat} LTR-DsRed, Clone # 131: unstimulated cells (left panel) and PMA-stimulated cells (right panel).

Appendix Figure S4

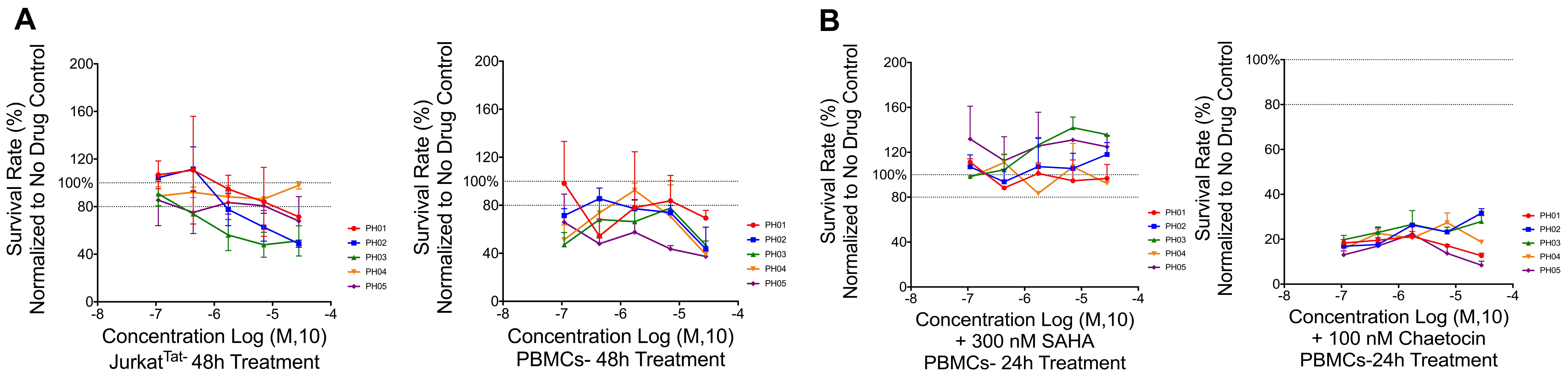

Appendix Figure S4. Effect of PH compounds on cell viability.

A Jurkat^{Tat} cells (left panel) and PBMCs from healthy donors (right panel) were treated with the indicated concentrations of the PH compounds for 48 hours.

B PH compounds in combination with 300 nM SAHA (left panel) and 100 nM Chaetocin (right panel) were tested on PBMCs from healthy donors. Cell viability was measured 24/48 hours later using an MTT assay. Results are normalized relative to untreated controls and presented as survival rate percentages.

Data information: Mean and SE for the results are determined from three biological replicates (n=3) and technical duplicates.

Appendix Figure S5

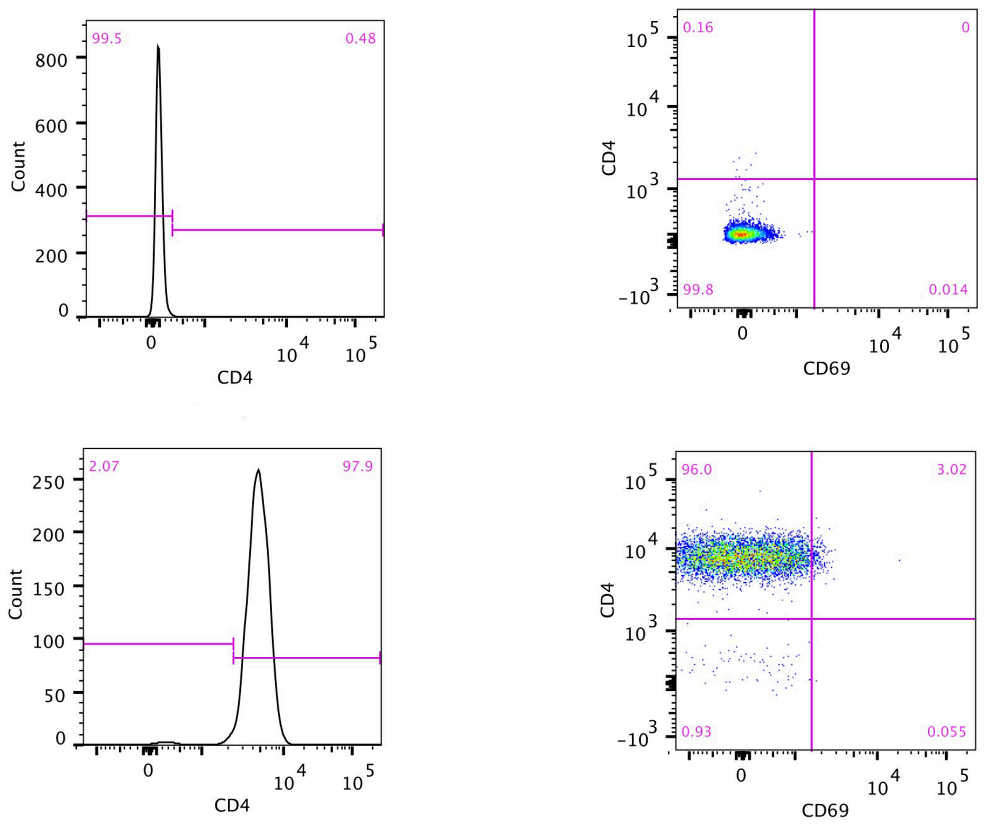

Appendix Figure S5. **Assessment of purity for resting T helper cells isolated patient samples.**
A CD4+ T cells isolated from whole blood of patient samples were stained with monoclonal antibodies against CD4+ and were analyzed by flow cytometry; approximately 98% of the isolated cells stained positive (lower panel) relative to unstained control cells (upper panel).
B Isolated CD4+ T cells from patient samples were stained with monoclonal antibodies against both CD4 and CD69 and analyzed by flow cytometry. Approximately 96% of the purified cells express CD4 but not CD69 (lower panel) compared to unstained control cells (upper panel).

Appendix Table S1

Figure	Comparison	*P-Value*
**3A**	No Drug Control vs. PMA	***P= *0.0008*
**4A**	PH01 vs. PH01+ SAHA	***P= 0.0007
	PH01 vs. PH01 + Chaetocin	*P= 0.169
	PH01 vs. PH01 + PEP005	****P< 0.0001
	SAHA vs. PH01 + SAHA	***P= 0.0002
	Chaetocin vs. PH01 + Chaetocin	**P= 0.0065
	Ionomycin vs. PH01 + Ionomycin	*P= 0.0325
	PEP005 vs. PH01 + PEP005	****P< 0.0001
**4B**	PH02 vs. PH02 + SAHA	**P= 0.0049
	PH02 vs. PH02 + Chaetocin	**P= 0.0057
	PH02 vs. PH02 + Ionomycin	*P= 0.0412
	PH02 vs. PH02 + PEP005	****P< 0.0001
	SAHA vs. PH02 + SAHA	*P= 0.0136
	Chaetocin vs. PH02 + Chaetocin	*P= 0.0197
	PEP005 vs. PH02 + PEP005	****P< 0.0001

Figure	Comparison	*P-Value*
**4C**	PH03 vs. PH03 + Chaetocin	*$P = 0.0205$
	PH03 vs. PH03 + Ionomycin	*$P = 0.0196$
	PH03 vs. PH03 + PEP005	****$P < 0.0001$
	PEP005 vs. PH03 + PEP005	****$P < 0.0001$
**4D**	PH04 vs. PH04 + Chaetocin	**$P = 0.0055$
	PH04 vs. PH04 + Ionomycin	*$P = 0.0176$
	PH04 vs. PH04 + PEP005	****$P < 0.0001$
	Chaetocin vs. PH04 + Chaetocin	**$P = 0.0077$
	PEP005 vs. PH04 + PEP005	****$P < 0.0001$
**4E**	PH05 vs. PH05 + PEP005	****$P < 0.0001$
	PEP005 vs. PH05 + PEP005	****$P < 0.0001$
**4F**	PH Compounds alone vs. PH Compounds + 1 nM PEP005	**$P = 0.0022$
	PH Compounds alone vs. PH Compounds + 2 nM PEP005	***$P = 0.0004$
	PH Compounds alone vs. PH Compounds + 4 nM PEP005	***$P = 0.0008$

Figure	Comparison	*P-Value*
**5C- Left Panel**	No Drug Control vs. PMA	****P< 0.0001
	No Drug Control vs. PH02 (-4.8 (Concentration Log (M,10))	****P< 0.0001
	No Drug Control vs. PH02 (-4.5 (Concentration Log (M,10))	****P< 0.0001
**5C- Right Panel**	PEP005 vs. PH02 (-4.8) + PEP005	****P< 0.0001
	PEP005 vs. PH02 (-4.5) + PEP005	****P< 0.0001
**5D- Left Panel**	No Drug Control vs. PMA	****P< 0.0001
	No Drug Control vs. PH02 (-4.8 (Concentration Log (M,10))	****P< 0.0001
	No Drug Control vs. PH02 (-4.5 (Concentration Log (M,10))	****P< 0.0001
**5D- Right Panel**	PEP005 vs. PH02 (-4.8) + PEP005	****P< 0.0001
	PEP005 vs. PH02 (-4.5) + PEP005	****P< 0.0001
**6A- Right Panel**	No Drug Control vs. PH02 + PEP005	****P< 0.0001
	PH02 vs. PH02 + PEP005	***P= 0.0003
	PEP005 vs. PH02 + PEP005	***P= 0.0005

Figure	Comparison	P-Value
**9**	No Drug Control vs. PMA	****$P < 0.0001$
	No Drug Control vs. 2 nM PEP005	*$P = 0.0469$
	No Drug Control vs. PH02 + 2 nM PEP005	**$P = 0.0087$
	No Drug Control vs. 4 nM PEP005	****$P < 0.0001$
	No Drug Control vs. PH02 + 4 nM PEP005	****$P < 0.0001$
**10A**	PH02 vs. PH02 + PEP005	*$P = 0.0500$
	PEP005 vs. PH02 + PEP005	**$P = 0.0022$
**10B**	No Drug Control vs. PMA	****$P < 0.0001$
	No Drug Control vs. PH02 + PEP005	***$P = 0.0004$
	PH02 vs. PH02 + PEP005	**$P = 0.0040$
	PEP005 vs. PH02 + PEP005	***$P = 0.0005$
**Appendix Figure S2A**	PH Compounds alone vs. PH Compounds + Chaetocin	*$P = 0.0214$
	PH Compounds alone vs. PH Compounds + Ionomycin	*$P = 0.0217$
	PH Compounds alone vs. PH Compounds + PEP005	***$P = 0.0009$

Figure	Comparison	*P-Value*
**Appendix Figure S2B**	PH Compounds alone vs. PH Compounds +1 nM PEP005	***P*= 0.0022
	PH Compounds alone vs. PH Compounds + 2 nM PEP005	****P*= 0.0004
	PH Compounds alone vs. PH Compounds + 4 nM PEP005	***P*= 0.0008

Appendix Table S1. List of exact P-values determined from statistical tests.
The exact P-values obtained from statistical tests stated in the figure legends are indicated.

Appendix Table S2

Name	Analogs	Vendor	Compound ID	Compound Name	M.W	Polar Surface Area (PSA)	2D Similarities to the Parental Structure
PH02	$PH02_a$	Chembridge	7111897	N-(2-methoxyphenyl)-N-[(3-oxo-1-benzothien-2(3H)-ylidene)methyl]acetamide	325	46.6	95%
	$PH02_b$		7113263	(2-methoxyphenyl)[(3-oxo-1-benzothien-2(3H)-ylidene)methyl]formamide	311	46.6	90%
	$PH02_c$		6568745	2-methyl-5-(3-nitrobenzoyl)-1H-isoindole-1,3(2H)-dione	310	97.6	80%
	$PH02_d$		6566663	2-methyl-5-(4-nitrophenoxy)-1H-isoindole-1,3(2H)-dione	298	89.8	75%
	$PH02_e$		6854909	methyl 2-(5-{[3-(4-methoxyphenyl)-4-oxo-2-thioxo-1,3-thiazolidin-5-ylidene]methyl}-2-furyl)benzoate	452	69.0	75%

Appendix Table S2. Summary of PH02 analog structures.
Five analogs associated to the PH02 were examined using the LTR-luciferase cell line.

Appendix Table S3

# of CD4+ T cells pools	Patients included in each pool
1	P.1, P.2, P.3, P.4, P.05, P.06, P.07, P.08, P.09, P.10, P.11, P.12, P.13, P.14, P.15, P.16, P.17, P.18

Appendix Table S3. Aviremic HIV-1 infected patients used in this study.
Pool of resting CD4+ T cells purified from the number of HIV-1-infected patients on HAART listed in the table. A single point concentration (30μM) of the identified compounds was initially tested on these purified rCD4+ T cells.

Appendix Table S4

# of CD4+ T cells pools	Patients included in each pool
1	P.19, P.20, P.21, P.22
2	P.23, P.24
3	P.25, P.26, P.27, P.28

Appendix Table S4. Aviremic HIV-1 infected patients used in this study.
Three pools of CD4+ T cells (n=3), including the numbers of different HIV-1-infected patient samples are served as three biological replicates to measure the intracellular HIV mRNAs after indicated drug treatments.

Appendix Table S5

# of CD4+ T cells pools	Patients included in each pool
1	P.29, P.30, P.31
2	P.32, P.33
3	P.34, P.35
4	P.36, P.37, P.38
5	P.39, P.40, P.41

Appendix Table S5. Aviremic HIV-1 infected patients used in this study.
Five pools (n=5) of CD4+ T cells purified from HIV-1-infected patients are indicated in the table. These five pools served as five distinct biological replicates used in the qVOA to measure the frequency of latently HIV-1-infected cells carrying replication-competent provirus after 24 h drug treatment.
