# Supplementary material for: Compounds producing an effective combinatorial regimen for disruption of HIV‐1 latency
Source: EMBO Mol Med. 2017 Dec 15;10(2):160–74. doi: 10.15252/emmm.201708193 (PMC5838563; doi:10.15252/emmm.201708193)
Supplement: Supplementary file 2 — Expanded View Figures PDF [file EMMM-10-160-s002.pdf]

Expanded View Figures

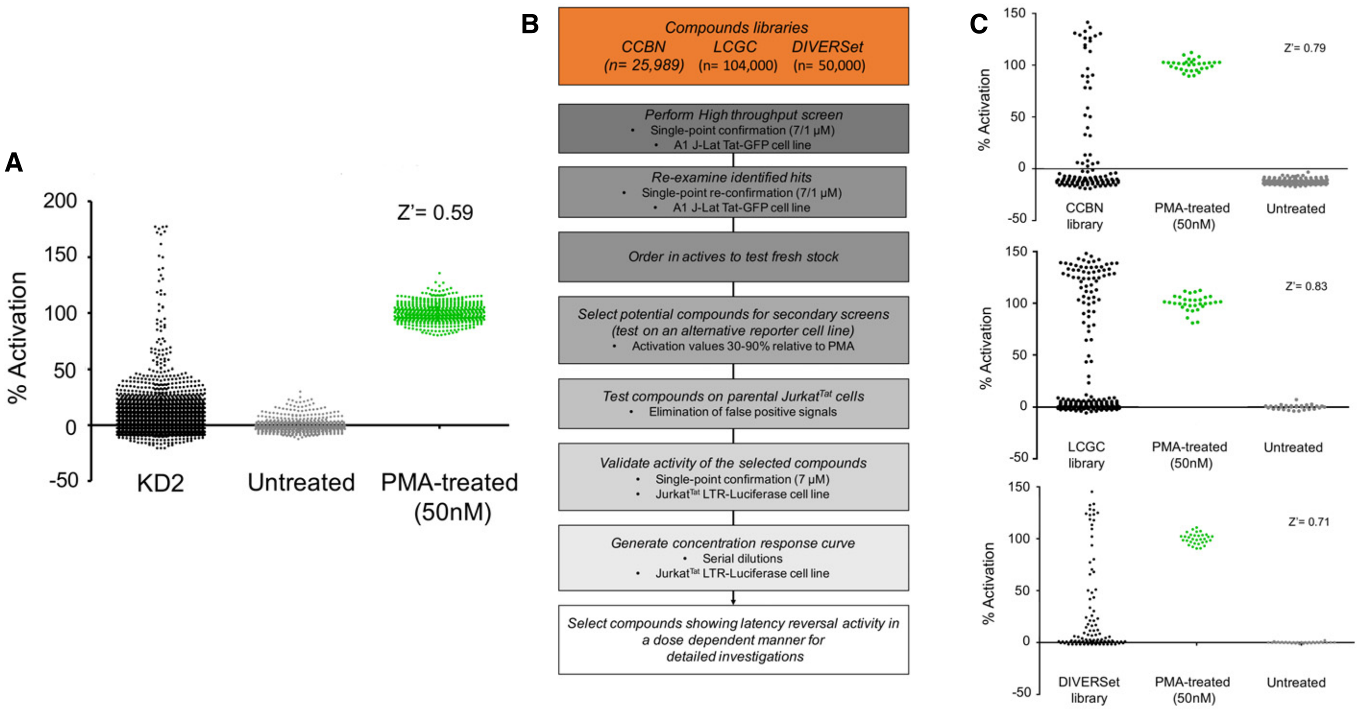

Figure EV1. Development of a high-throughput screening assay for HIV-1 latency-reversing activities.

A The bioactive library (KD2) was screened in 384-well format for latency-reversing activity using the A1 J-Lat cell line as described in Materials and Methods. The distribution of GFP expression produced by treatment with the compounds is shown as percentage activation relative to control samples treated with PMA for 24 h. The pilot screen produced a Z' factor higher than 0.5, suggesting that the assay was sufficiently robust for larger screens.

B Overview of the high-throughput screening plan and validation with secondary assays. Three compound libraries encompassing 180,000 small molecules were subjected to screens for disruption of HIV-1 latency at a concentration of 7/1 µM. Activity of compounds producing GFP expression upon re-assay at the same single concentration was re-ordered from their respective vendors and assayed using the LTR-luciferase cell line to confirm dose–response effects.

C The actives, identified in the first round of screening, were retested in the same format described above. In the second round of screening, a smaller number of those actives were reconfirmed and selected for further analyses.

Data information: Results are determined from three biological replicates (*n* = 3).

 © 2017 The Authors

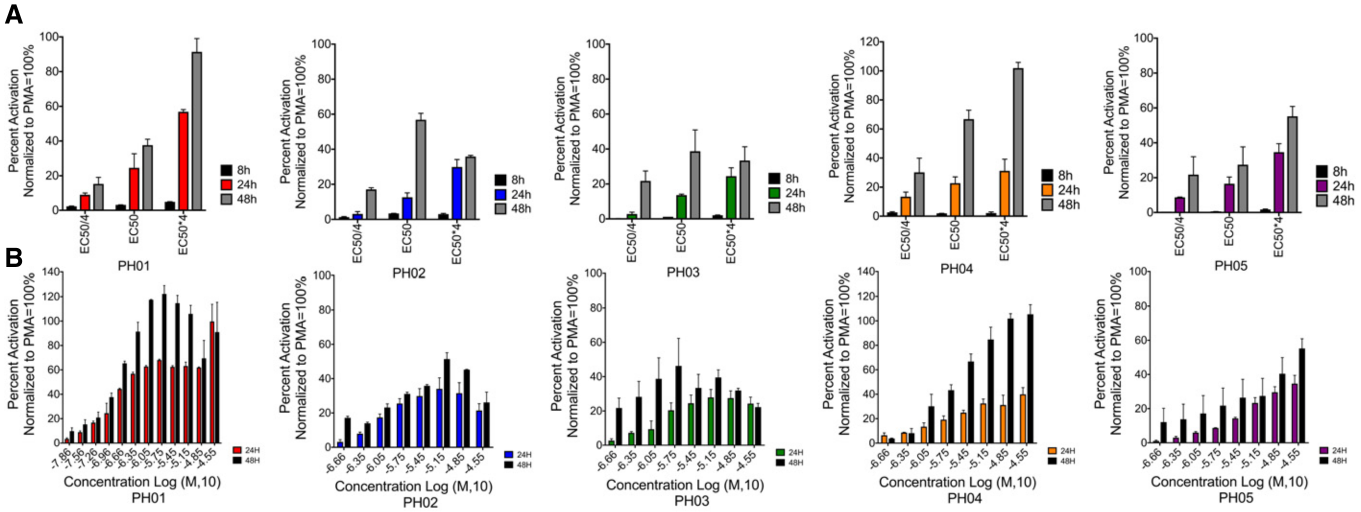

Figure EV2. Latency-reversal activity of the PH compounds at various times.

A Jurkat^{Tat} LTR-luciferase cells were treated with the EC50/4, EC50, and EC50*4 of PH01–PH05 for the indicated time points. Luciferase activity was measured post-treatment, and the results are presented as percent activation relative to results from PMA treatment.

B Activity of compounds PH01–PH05 on Jurkat^{Tat} LTR-luciferase cells treated for 24 and 48 h are compared. Concentrations of the compounds are indicated.

Data information: Mean and SE for the results are determined from three biological replicates (n = 3) and technical duplicates.

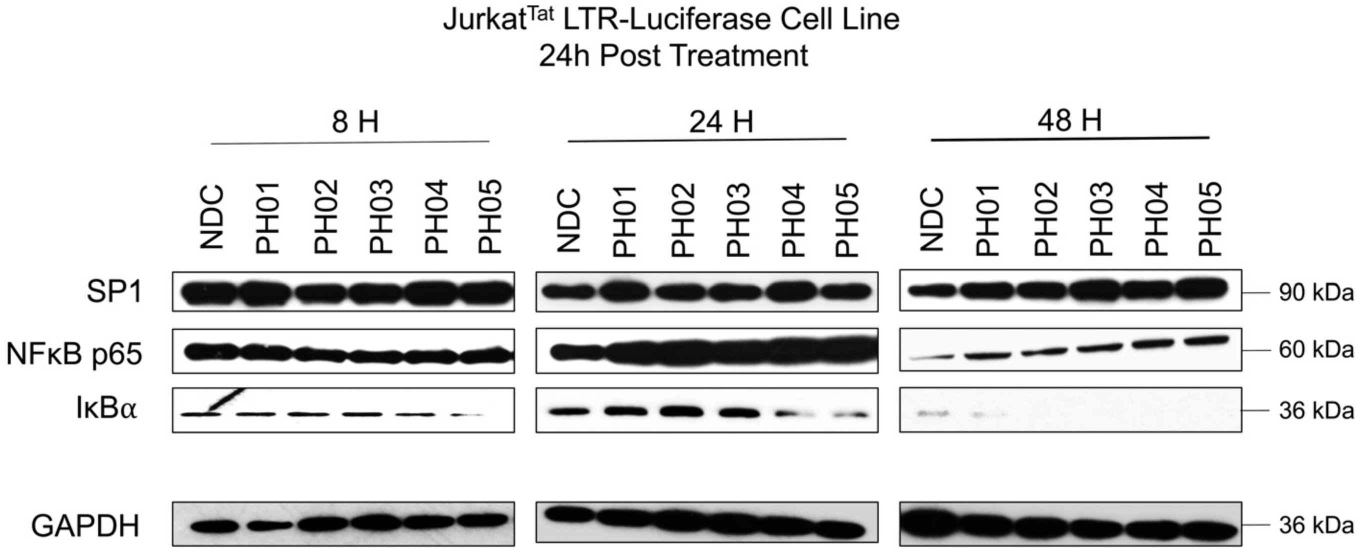

Figure EV3. Effect of PH compounds on SP1, NF-κB p65, and IκBα expression.

Jurkat^{Tat} LTR-Luciferase cells were treated with a single concentration (3 μM) of each PH compound for the indicated time points. Whole-cell protein extract was separated by SDS–PAGE, transferred to nitrocellulose membrane, and blotted with antibodies against SP1, NF-κB p65, and IκBα. GAPDH was used as the loading control.

Source data are available online for this figure.

© 2017 The Authors

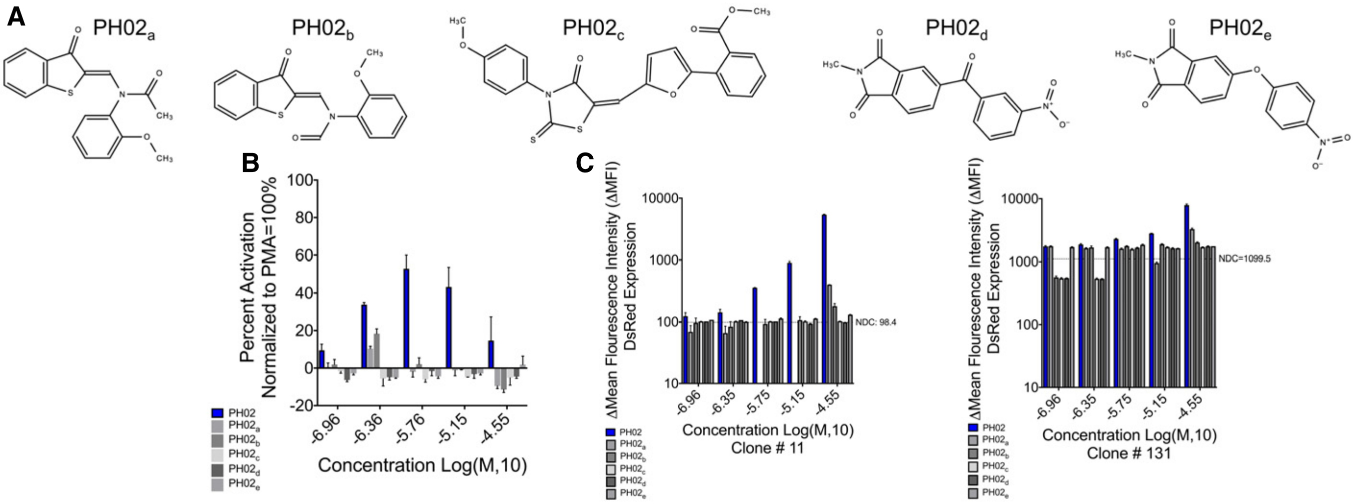

Figure EV4. Ability of PH02-associated analogs to reactivate HIV-1 LTR expression.

A Illustration of the chemical structures associated with the PH02 analogs, referred to as $PH02_{a–e}$, examined on the *in vitro* reporter cell lines for structural optimization.

B, C Jurkat^{Tat} LTR-luciferase cell line (B), Jurkat^{Tat} LTR-DsRed clone 11 (C, left panel), and clone 131 (C, right panel) were treated with the indicated concentrations of PH02-associated analogs. Luciferase activity or overall DsRed expression was measured 24 h post-treatment. Results are presented as percent activity relative to the positive control (PMA-treated cells) or Δ mean fluorescence intensity (ΔMFI) of DsRed expression, respectively. Mean and SE for the results are determined from three biological replicates (n = 3) and technical duplicates.

© 2017 The Authors

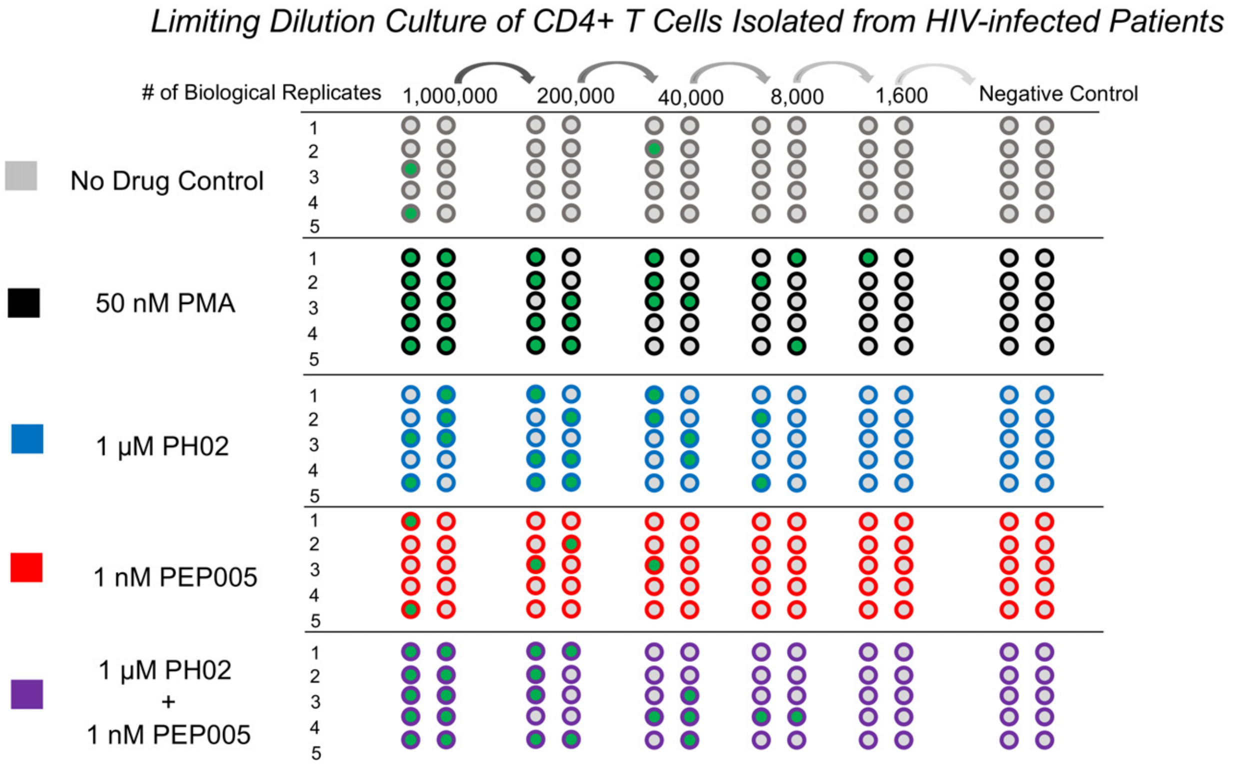

Figure EV5. Assay for reactivation of HIV-1 from latently infected cells using a quantitative viral outgrowth assay.

Resting $CD4^+$ T cells from patient samples were serially diluted in 6- or 24-well plates in duplicate (top) and treated as indicated (left). After 24 h, MOLT4/CCR5 cells were added to the wells, and the cultures incubated a further 14 days. HIV-1 RNA was detected in filtered culture supernatants using RT–qPCR, and wells producing the specific PCR product are indicated in green. The frequency of latently infected cells where viral replication was reactivated by treatment was estimated using the IUPMStats V1.0 infection frequency calculator (http://silicianolab.johnshopkins.edu), which generates a maximum-likelihood estimation (MLE) for latently infected cells.

© 2017 The Authors
