# Supplementary material for: Compounds producing an effective combinatorial regimen for disruption of HIV‐1 latency
Source: EMBO Mol Med. 2017 Dec 15;10(2):160–74. doi: 10.15252/emmm.201708193 (PMC5838563; doi:10.15252/emmm.201708193)
Supplement: Supplementary file 3 — Source Data for Expanded View [file EMMM-10-160-s004.pdf]

Fig EV3

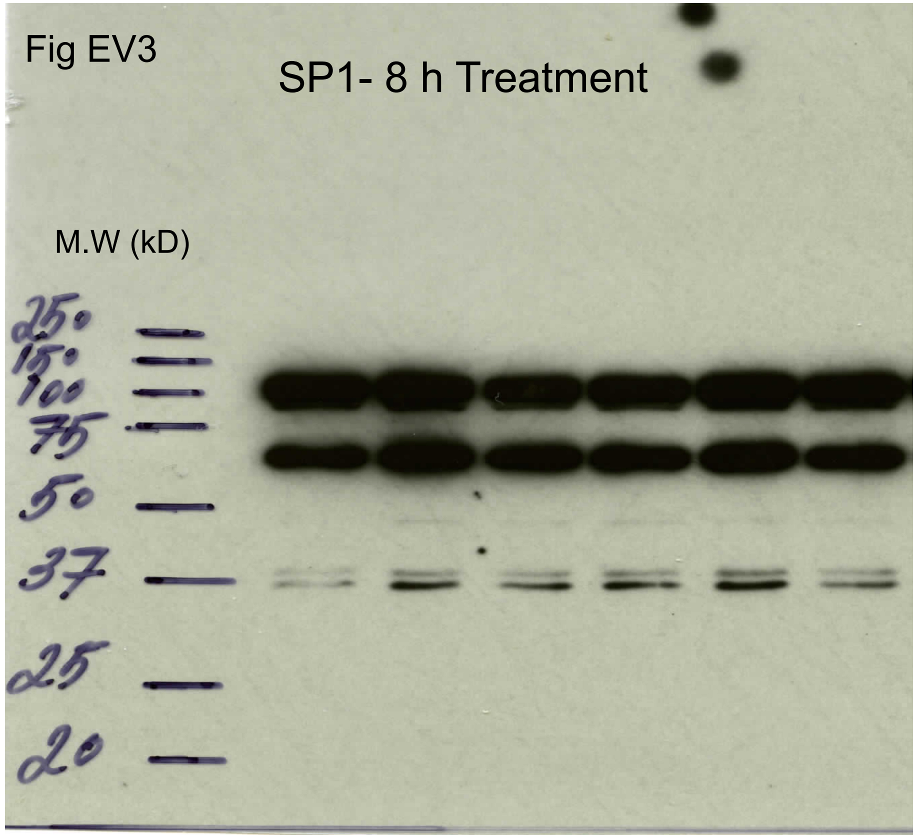

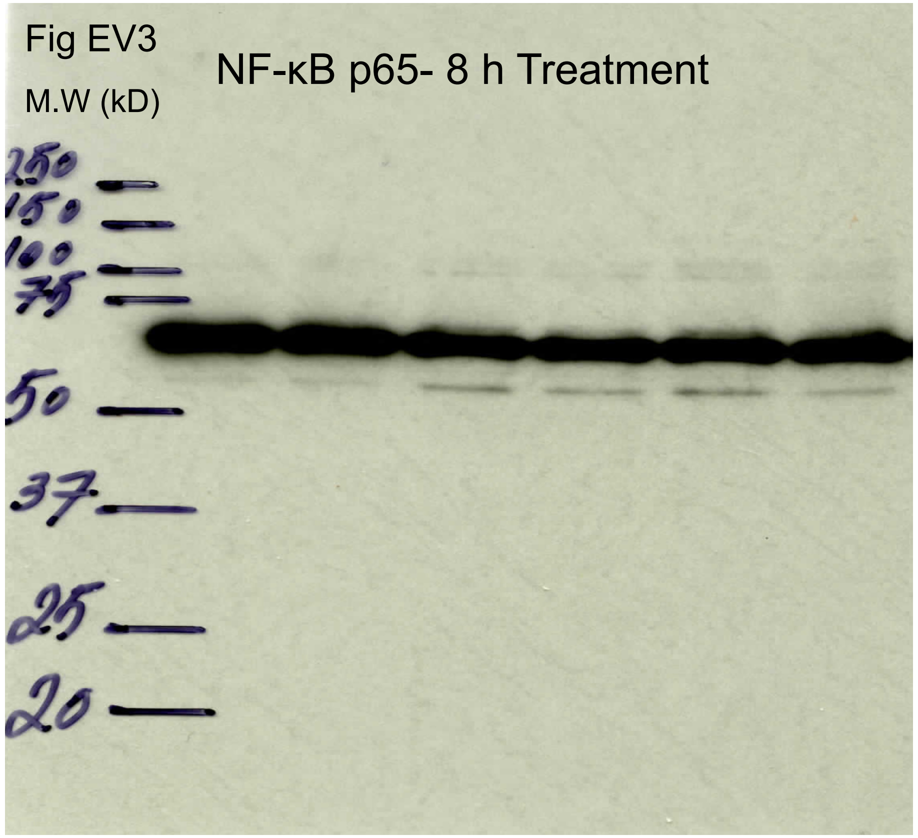

Fig EV3
M.W (kD)
NF-κB p65- 8 h Treatment
250
150
100
75
50
37
25
20

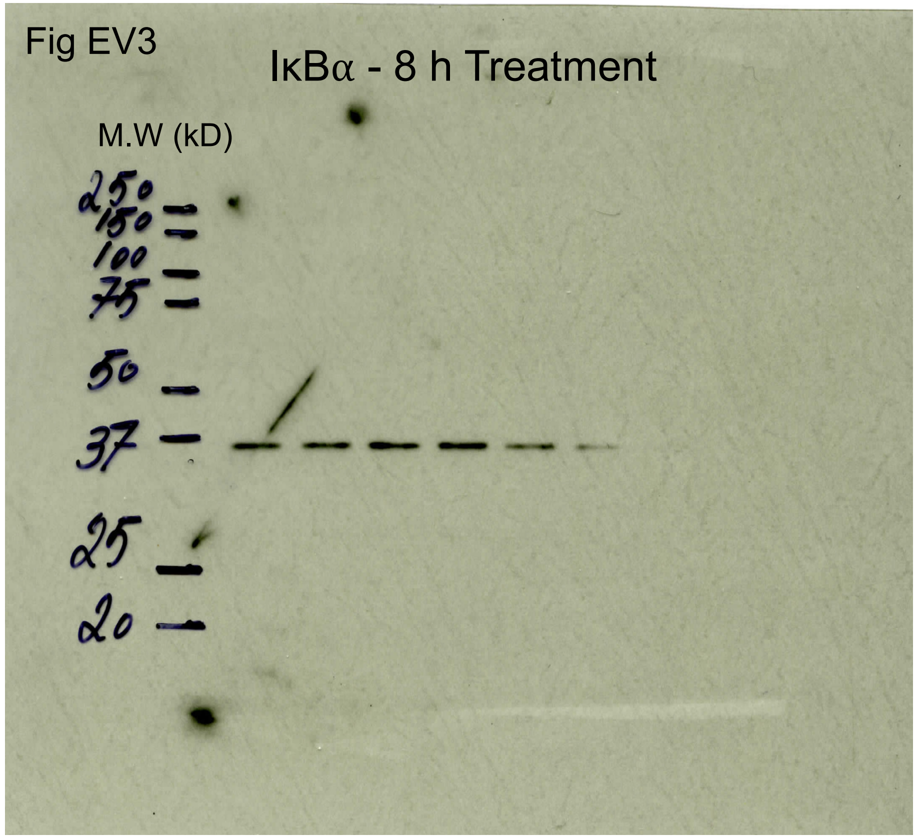

Fig EV3
IκBα - 8 h Treatment
M.W (kD)
250
150
100
75
50
37
25
20

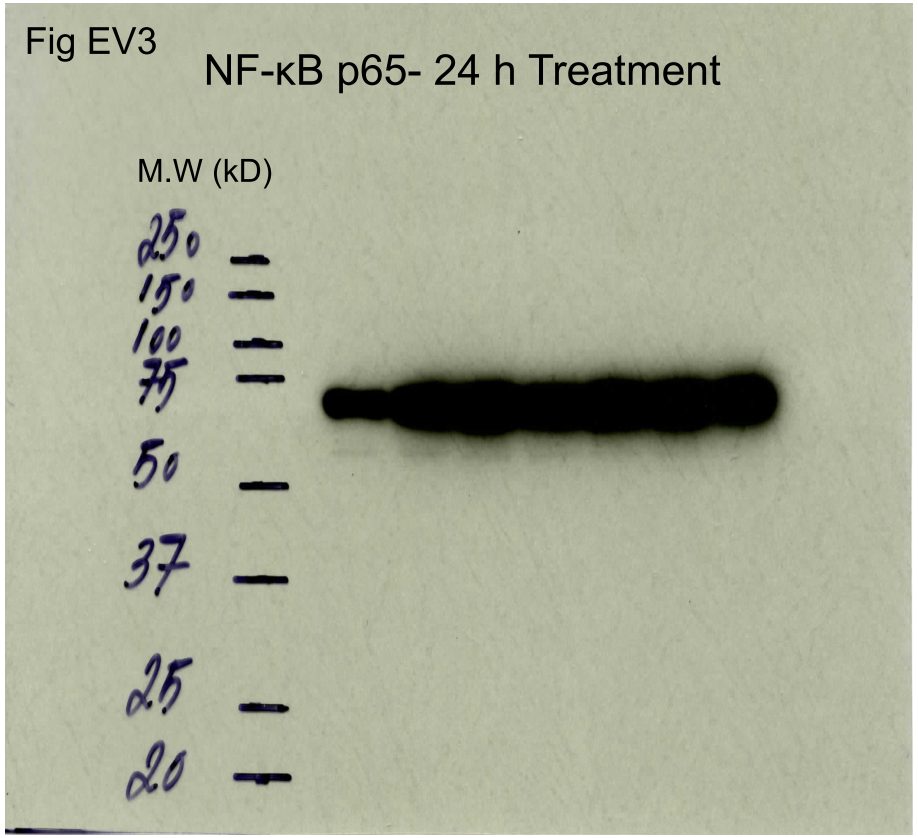
Fig EV3
NF-κB p65- 24 h Treatment
M.W (kD)
250
150
100
75
50
37
25
20

Fig EV3

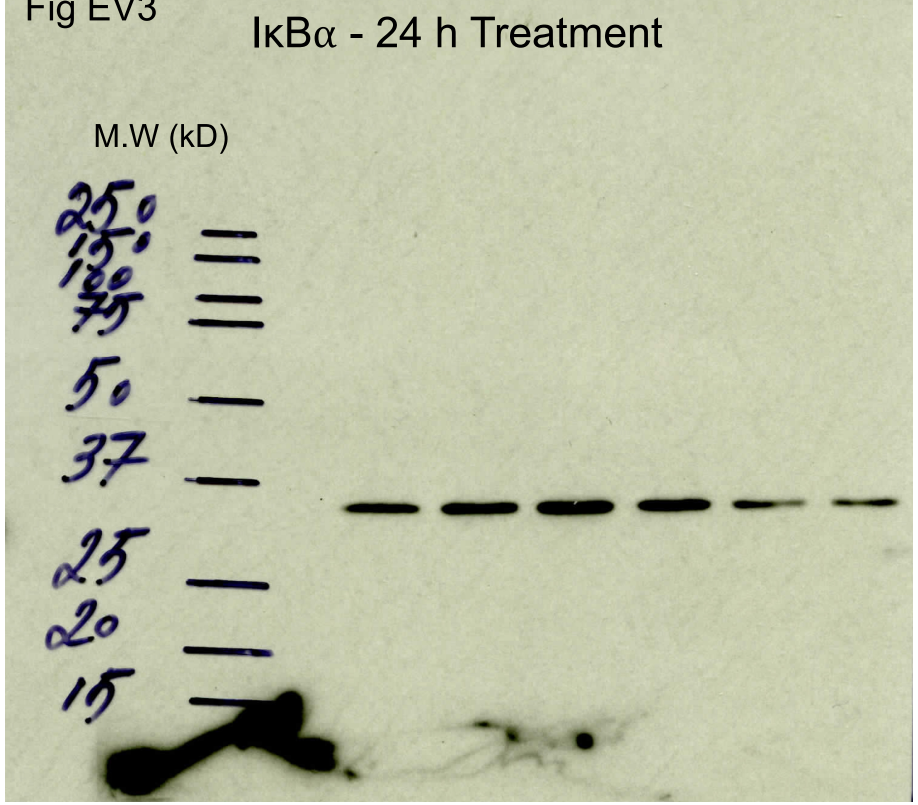

Fig EV3

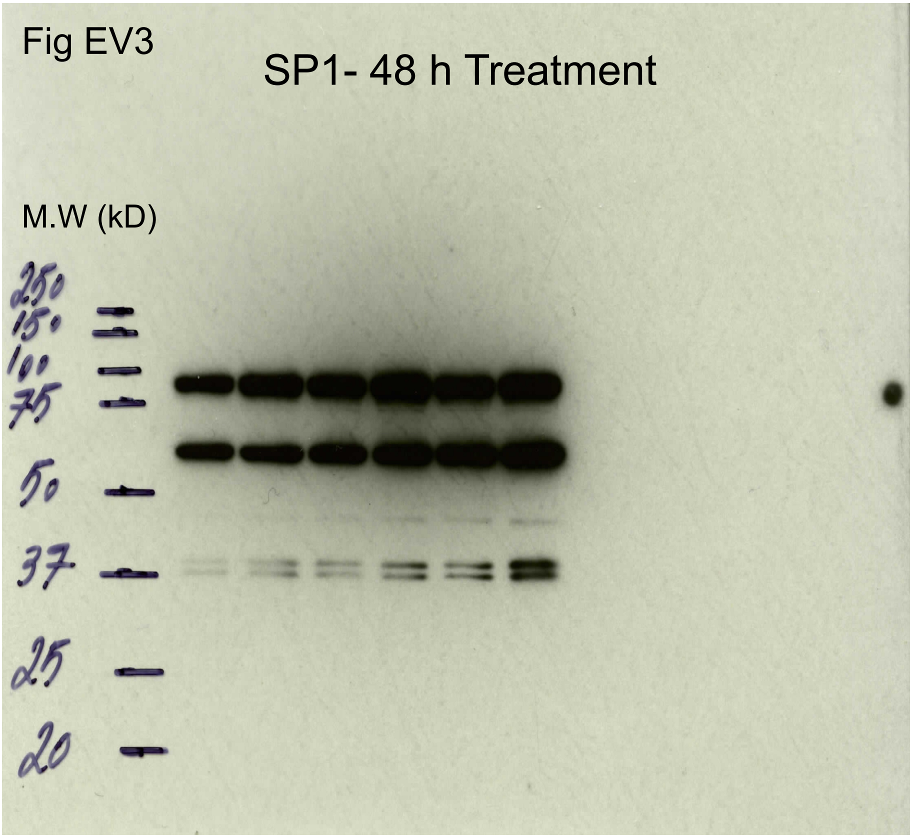

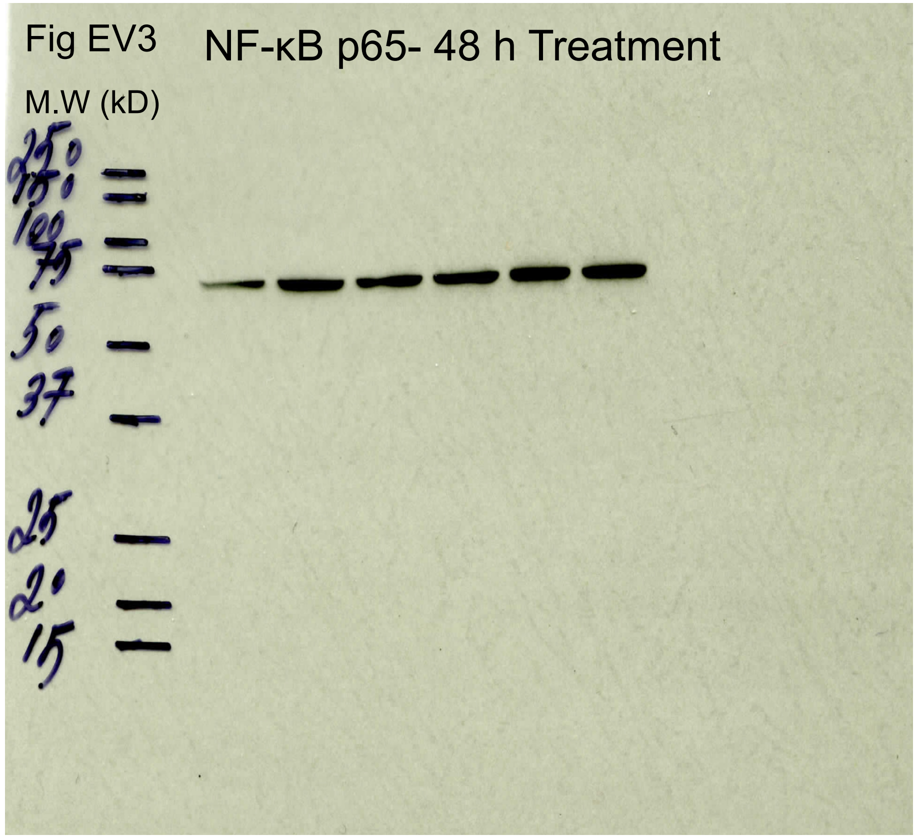
Fig EV3
NF-κB p65- 48 h Treatment
M.W (kD)
250
150
100
75
50
37
25
20
15

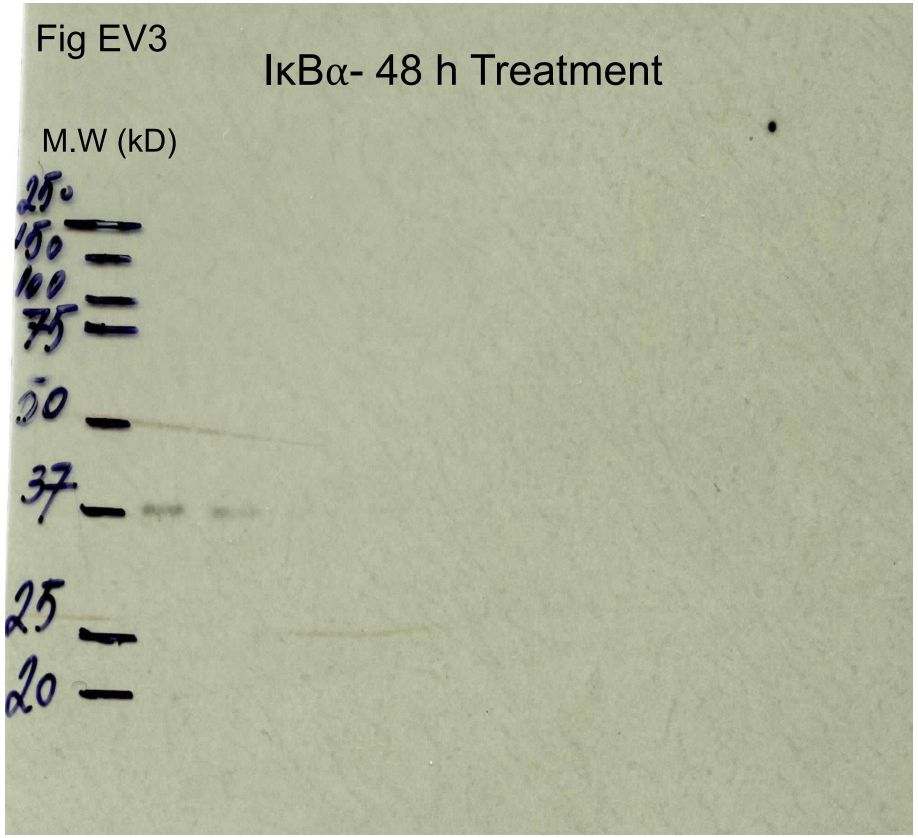
Fig EV3
IκBα- 48 h Treatment
M.W (kD)
250
150
100
75
50
37
25
20
