# Supplementary figures and images for: Compounds producing an effective combinatorial regimen for disruption of HIV‐1 latency
Source: EMBO Mol Med. 2017 Dec 15;10(2):160–74. doi: 10.15252/emmm.201708193 (PMC5838563; doi:10.15252/emmm.201708193)

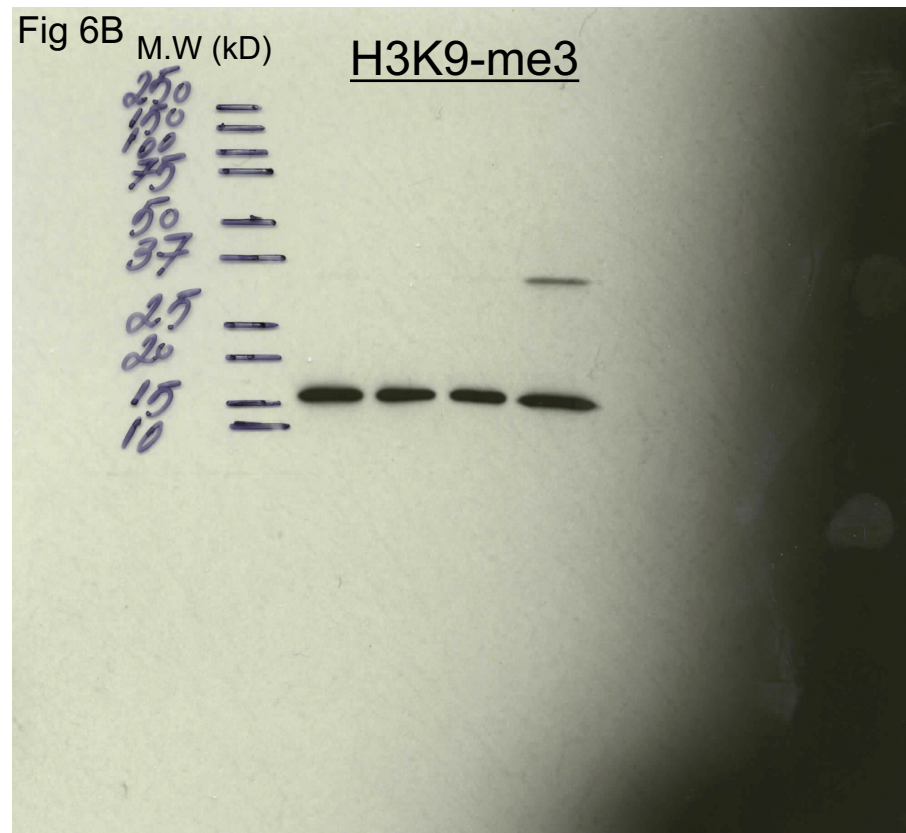

Fig 6B

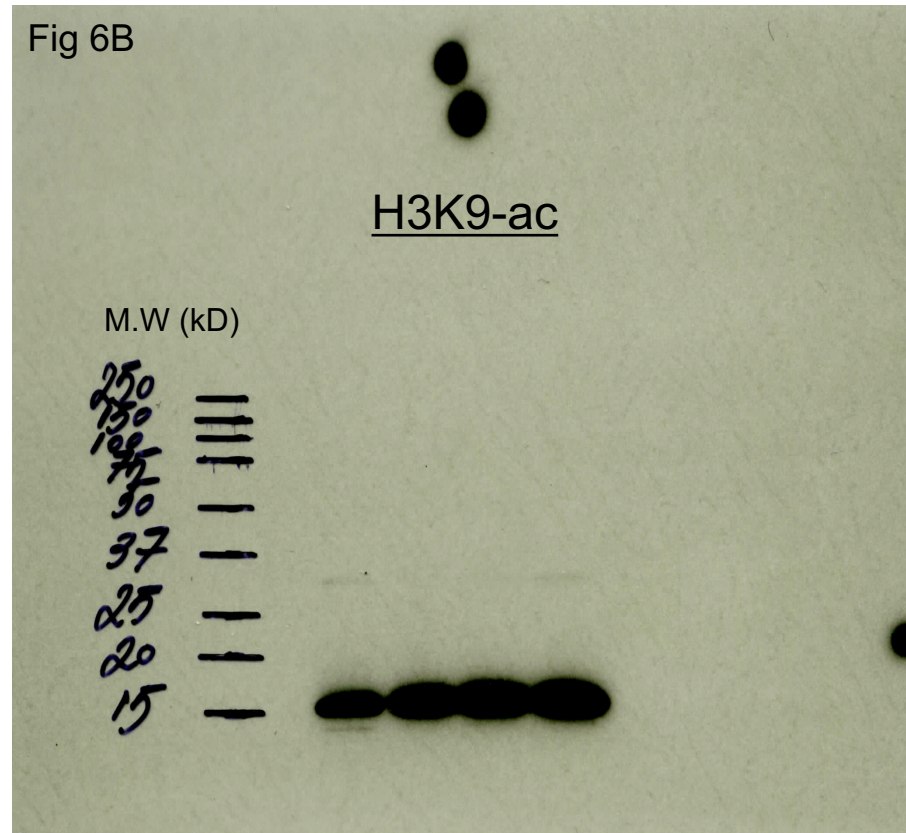

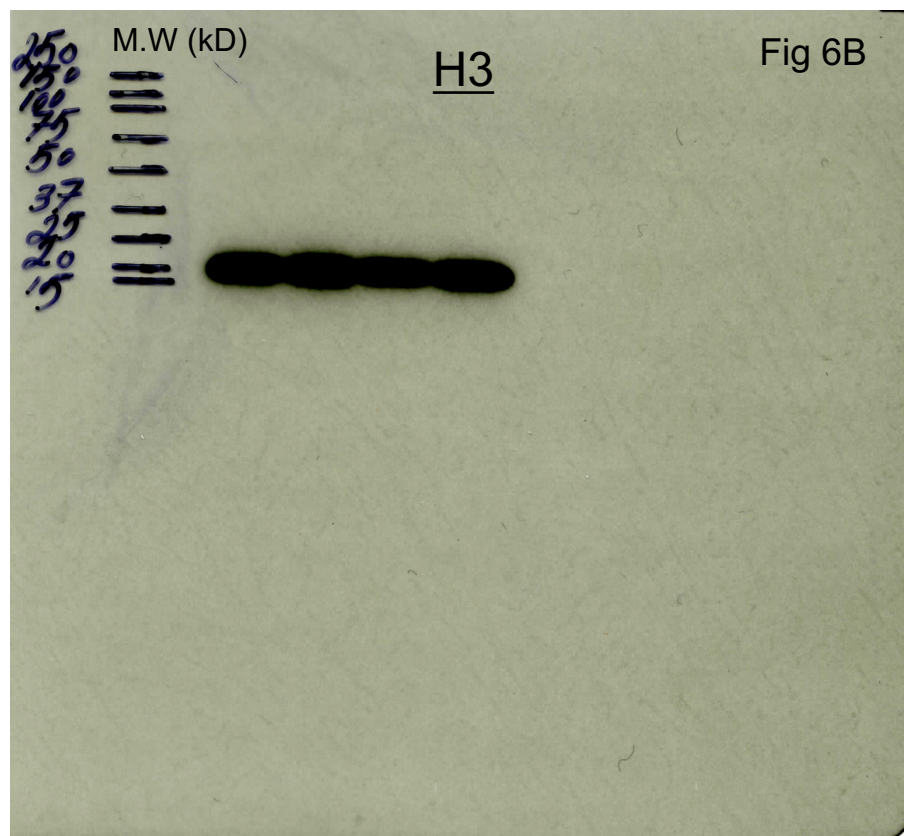

Fig 6B

SP1

M.W (kD)

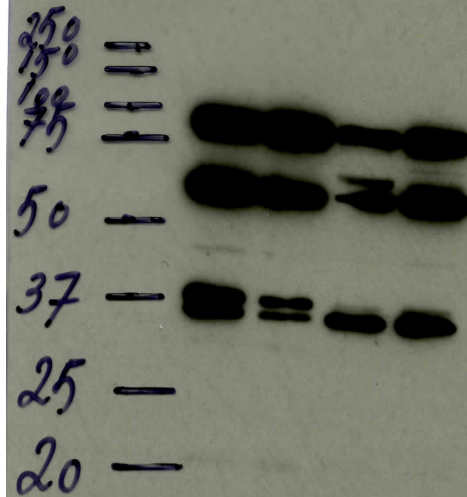

Fig 6B

NF- $\kappa$ B p65

M.W (kD)

250 —  
150 —  
100 —  
75 —  
50 —  
37 —  
25 —  
20 —

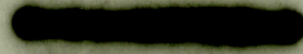

Fig 6B

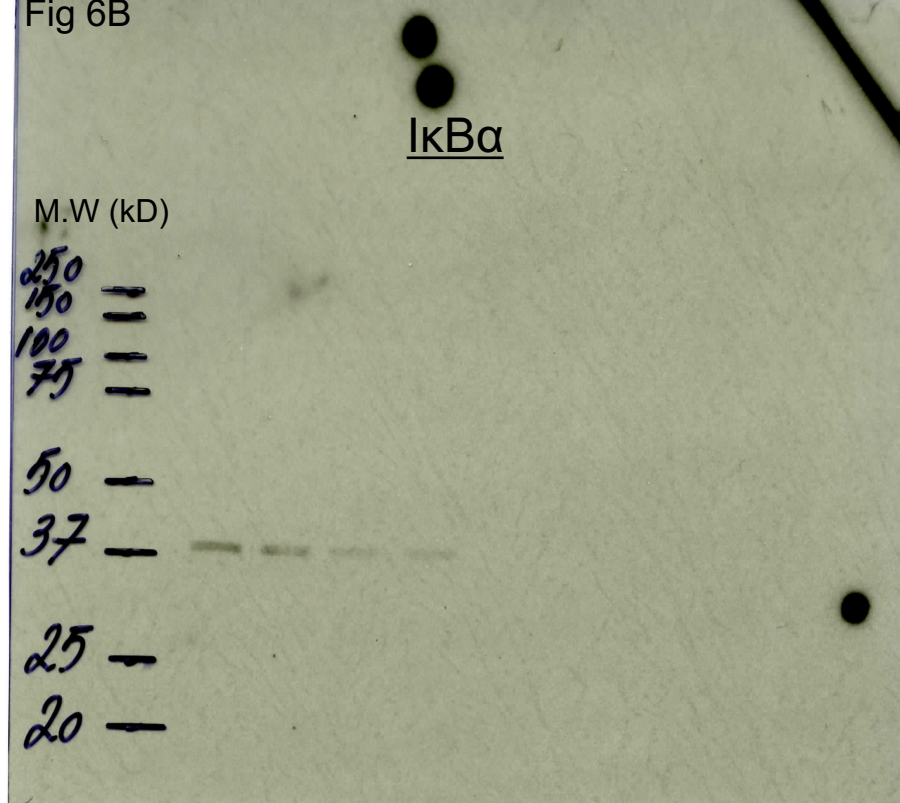

Supplement: Supplementary file 5 — Source Data for Figure 6 [file EMMM-10-160-s003.pdf]
